# Single Fluorescence Channel-based Multiplex Detection of Avian Influenza Virus by Quantitative PCR with Intercalating Dye

**DOI:** 10.1038/srep11479

**Published:** 2015-06-19

**Authors:** Christian D. Ahberg, Andreas Manz, Pavel Neuzil

**Affiliations:** 1 Kist-Europe, Campus E7.1, Saarbruecken, Germany D-66123; 2 Brno University of Technology, Technická 10, Brno, Czech Republic; 3 Institute of Bioengineering and Nanotechnology, 31 Biopolis Way, The Nanos, #04-01, 138669 Singapore

## Abstract

Since its invention in 1985 the polymerase chain reaction (PCR) has become a well-established method for amplification and detection of segments of double-stranded DNA. Incorporation of fluorogenic probe or DNA intercalating dyes (such as SYBR Green) into the PCR mixture allowed real-time reaction monitoring and extraction of quantitative information (qPCR). Probes with different excitation spectra enable multiplex qPCR of several DNA segments using multi-channel optical detection systems. Here we show multiplex qPCR using an economical EvaGreen-based system with single optical channel detection. Previously reported non quantitative multiplex real-time PCR techniques based on intercalating dyes were conducted once the PCR is completed by performing melting curve analysis (MCA). The technique presented in this paper is both qualitative and quantitative as it provides information about the presence of multiple DNA strands as well as the number of starting copies in the tested sample. Besides important internal control, multiplex qPCR also allows detecting concentrations of more than one DNA strand within the same sample. Detection of the avian influenza virus H7N9 by PCR is a well established method. Multiplex qPCR greatly enhances its specificity as it is capable of distinguishing both haemagglutinin (HA) and neuraminidase (NA) genes as well as their ratio.

The influenza A virus is composed of eight RNA segments of negative-sense single-stranded RNA where segment 4 encodes the haemagglutinin (HA) gene, and segment 6 the neuraminidase (NA) gene. In order to increase specificity of H7N9 detection, besides the detection of the virus itself, one should also detect both HA and NA individually as well as their ratio. PCR is the method of choice to detect the virus as it can deliver results in tens of minutes compared to traditional methods such as ELISA where the testing takes a few days.

The polymerase chain reaction (PCR) was invented in 1985^1^,^2^ to amplify double stranded DNA segments. Adding fluorogenic probe or DNA intercalating dyes (such as SYBR Green) allowed real-time^3^ reaction progress monitoring and extracting of quantitative information (qPCR)^4^,^5^. A typical PCR process consists of three steps conducted at different temperatures: denaturation at 95 °C, annealing at 50–60 °C and extension at 72 °C. Using the Taqman probe-based PCR system the annealing and extension steps are combined into one and performed at a temperature of about 60 °C^5^. The presence of end-point PCR products can be confirmed by agarose gel electrophoresis or by capillary electrophoresis (CE)^6^. However, an end-point measurement does not necessarily correlate with the original number of copies of the amplified DNA sequence. Real-time PCR allows precise quantitative information to be extracted from the exponential phase of the reaction^4^,^7^. The Taqman probe-based assay format, for example the FAM^TM^ (fluorophore) and TAMRA^TM^ (quencher) labeled probe, is specific to its target gene, whereas the SYBR Green-based format is non-specific. To determine the specificity of the PCR products using SYBR Green, a subsequent melting curve analysis^8^ (MCA) has to be implemented. MCA is often preferred over CE as it is performed in the PCR system immediately once the PCR is completed by sweeping samples’ temperature while monitoring amplitude of fluorescence and thus no sample manipulation is required.

Multiplex quantitative PCR (qPCR) methods based on Taqman probes as well as FRET-based systems have been demonstrated^9^. Currently, it is routinely done with probes, such as the popular FITC, JOEL, ROX and Cy5, using multiple optical channels. Each channel requires its corresponding light source, filter set and a detector. Can multiplex qPCR be conducted in one channel, while demultiplexing the results in real-time? Probe-based systems cannot be used as there is no technique capable of distinguishing the PCR products in a real time. On the other hand intercalating dye based PCR has shown promising results as the products can be demultiplexed using melting curve analysis (MCA) once the PCR is completed^10^. However, this method can determine serotype of the DNA or RNA but does not provide quantitative information. Continuous monitoring of the amplitude of the fluorescent signal is a powerful technique with a number of different applications^11^. One of them was product differentiation during PCR where a number of MCAs were performed individually after several thermal cycles of PCR^12^.

In this contribution we propose and demonstrate a method to dynamically extract melting curves within each thermal cycle of a real-time PCR based on a single intercalating dye. This was accomplished by processing captured data without changing the PCR protocol, resulting in a set of 40 MCAs. From this set we were able to demultiplex quantitative data for different segments of DNA. The proposed method allows multiplex internal positive controls using a single intercalating dye and is an alternative for probe based systems used to detect HA to NA gene ratios. It employs only a single fluorescent optical channel.

Typical MCA for intercalating dye-based PCR is performed by scanning the temperature of the sample at the rate of 1 °C/s or lower while recording the corresponding amplitude of fluorescence signal. The slow scanning rate is an important factor as every system with temperature control such as PCR exhibits a delay between temperature at the sensor and the sample. PCR systems can have a delay as long as a few seconds or more which is why slow temperature scanning for precise determination of a melting curve is essential.

During PCR the sample is cycled between different temperatures. Especially interesting is the transition from extension to denaturation as it covers the range of expected melting temperatures. All that is needed, then, is to detect the fluorescence amplitude from the sample and determine the corresponding temperature during this phase.

The first task has been demonstrated earlier by continuous fluorescence monitoring during the PCR cycling^11^. However the precise temperature monitoring of the sample during temperature transitions is not straightforward as there is discrepancy between sample and heater temperature as explained above. Is there another way to monitor actual sample temperature during ramping from annealing step to denaturation step?

We believe there is a method to measure the sample temperature with high precision. This temperature is given by a thermal control system typically using proportional integrated derivative (PID) method of feedback, the thermal mass of the sample *H* and the thermal conductance *G* between the heating block and the sample. The time constant *τ* of the temperature delay is given by *τ* = *H*/*G*. Values of *H*, *G* and PID constants do not change during the PCR process so the temperature profile of the sample is a repetitive function of time during the PCR cycling. This implies that it is sufficient to determine the temperature profile of the sample only during a single cycle. We have used a modified version (see Fig. 1) of the virtual reaction chamber (VRC) system for this experiment^13^. The heat transfer and the sample temperature profile was simulated by finite element analysis (FEA) and the results were experimentally verified^14^. The model gave us a correlation between the temperature of the heater and the sample during transition from annealing step to denaturation step required for the MCA extraction. The real sample experiment exhibited faster response than the model probably due to convection in the aqueous sample decreasing the temperature gradient minimally in the transition period. This also provided a homogenous temperature distribution, crucial to the method, since it suppresses data smearing.

Here we introduce a method of multiplex qPCR using a single fluorescence channel in the presence of Eva-Green intercalator. It is based on recording both temperature *T* and fluorescence *F* as a function of time *t* and cycle number *n.* From both functions [*F* = f(*t,n*) and *T *= f(*t,n*)] we have then eliminated time and generated fluorescence as function of temperature [*F* = f(*T,n*)] with PCR cycle number as a parameter. We have thus performed a melting curve analysis (MCA) during each PCR cycle. Subsequently we plotted the first negative derivative of fluorescence with respect to temperature as a function of temperature −d*F*/d*T* = f(*T*). For each amplicon a distinct melting temperature was obtained, with its amplitude corresponding to its concentration at each cycle. PCR amplification curves were then created by plotting the amplitude of the respective amplicon as function of cycle number. This way we can quantitatively detect one or more different amplicons using only a single intercalating dye. We only need to perform continuous fluorescence detection at an acquisition rate at or above 1 kHz, thus conventional reagents and protocols can be still used.

The only prerequisite for the methods are a high enough data acquisition rate of fluorescence amplitude, a small sample volume and a sufficiently large difference in melting temperature of the genes for differentiation.

## Materials and Methods

### Preparation of PCR mixture and thermal cycling

PCR mixture consisted of 6μL Roche LightCycler TaqMan Master Mix (Roche Diagnostics, Germany), 1 μL 20X EvaGreen Dye in water (TATAA, Sweden) as well as forward and reverse primers^15^ (Table 1)(MWG Eurofines, Germany) at a final concentration of 1.8 μM. To this solution synthetic complementary DNA (cDNA) templates (ATG Biosynthesis, Germany) for haemagglutinin (HA) and neuraminidase (NA) for the avian influenza virus (H7N9) were added at different concentrations (see Table 1). Lastly the solution was adjusted to a volume of 20 μL using water obtained from a Milli-Q ProgradT3 column (Millipore, Germany).

**Figure 1:**
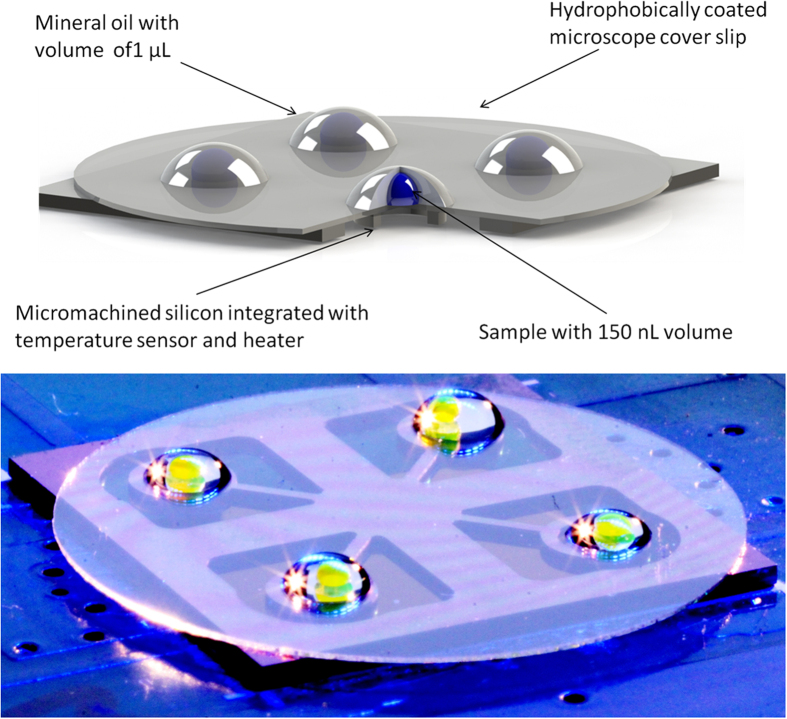
(**A**) Schematic diagram of the sample and heater. (**B**) Photograph of four VRCs on silicon chip with sample replaced with fluorescein solution for visualization purpose.

A droplet with a volume of 150 nL of the PCR solution was placed on a hydrophobically coated microscope glass cover slip and covered with 1.5 μL of mineral oil 9405 (Sigma Aldrich, Germany) thus forming a VRC. This glass was then placed on a micromachined silicon chip integrated with both a heater and a sensor, similar to a design shown earlier^13^. Here the silicon chip has a size of only 15 × 15 mm to better fit a handheld PCR device we are developing (see Fig. 1). The small thermal mass of the VRC together with the silicon heater resulted in heating rates >20 °C/s and similar cooling rates achieved only by passive cooling. The PCR protocol consisted of a hot start for 10 min at 95 °C followed by 40 cycles each consisting of two steps, denaturation for 5 s at 95 °C and annealing/extension for 30 s at 60 °C.

Fluorescence amplitude was continuously monitored using an Axiotron II microscope (Zeiss, Germany). We have used a blue LED model M470L3-C4 LED with principal wavelength of 470 nm (Thorlabs, Germany) for excitation. The LED was powered with square wave pulses at a frequency of 2710 Hz and a duty cycle of 5%. Light from the LED as well as emitted light from the specimen was filtered with a filter set model 49002 - ET - EGFP (FITC/Cy2) (Chroma Optical Corp, USA). Emitted light was captured by a PMT photosensor module H10722-20 (Hamamatsu Photonics K.K., Germany). The PMT signal was processed by a lock-in amplifier 7230 DSP (Ametek, USA) and its output recorded by a digital oscilloscope model DPO 3014 (Tektronix, Germany) with data rate of 2500 measurements per second. The temperature of the PCR chip was also recorded by the oscilloscope at the same data rate.

For comparison, samples were also analyzed using the Roche LightCycler Carousel-Based system (Roche Diagnostics, Germany) followed by a standard melting curve after thermal cycling.

### Data analysis

Captured fluorescence data were analyzed using a custom written Matlab-script. First the data were filtered by a fast Fourier transform filter (FFTF). Subsequently, the fluorescence signal was divided into the individual cycles based on the captured temperature profile. The fluorescence amplitude during transition from extension/annealing step to denaturation step was then extracted for each cycle and fluorescence as function of temperature was plotted with cycle number as a parameter. MCA was formed by numerical differentiation of the fluorescence with respect to temperature and its negative value was plotted as a function of temperature. The melting curve captured at the first cycle was subtracted from all melting curves to suppress a background fluorescence effect we have observed. This background fluorescence could be caused by autofluorescence from the adjacent printed circuit board as well as fluorescence of the temperature dependence of the fluorophore as well as unspecific binding of the dye onto single stranded DNA^16^. PCR amplification curves were created by plotting amplitudes at the MCA of each amplicon as a function of the cycle number.

### Results and Discussion

**Figure 2:**
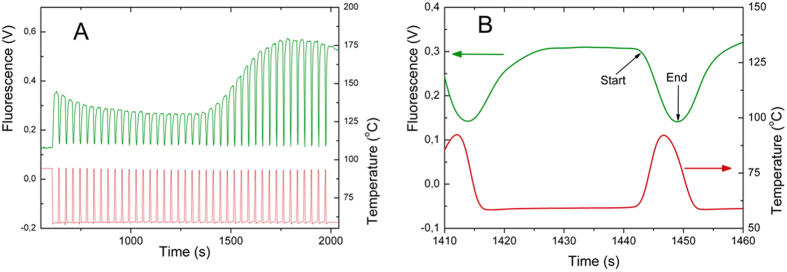
(**A**) Continuous fluorescence profile and corresponding temperature captured of entire PCR experiment. The 10 minutes of hot start is not completely shown, thermal cycling starts after 600 seconds. (**B**) Single PCR step number 23 showing fluorescence amplitude (green, left axis) and temperature (red, right axis). Start and end of data recording for MCA is shown by arrows.

We have run a 40 cycle PCR using the master mix manufacturer’s specifications. A typical fluorescence profile was obtained (see Fig. 2A). In Fig. 2B we show the single cycle number 23 to demonstrate the extracted data for the MCA.

**Figure 3:**
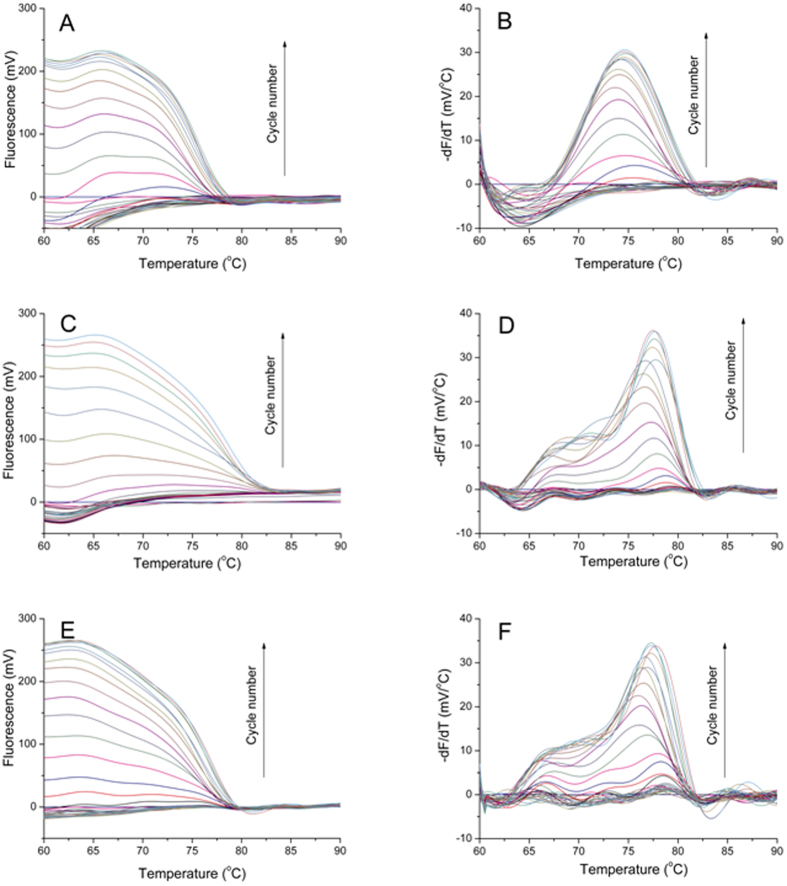
Melting curves (**A**, **C**, **E**) and corresponding to their first negative derivative (**B**, **D**, **F**) for all 40 cycles with cycle number as parameter. **A** and **B** are data from sample containing only HA. C and D are data from sample containing HA and twice more of NA. **E** and **F** are data from sample containing HA and ten times more NA.

**Figure 4:**
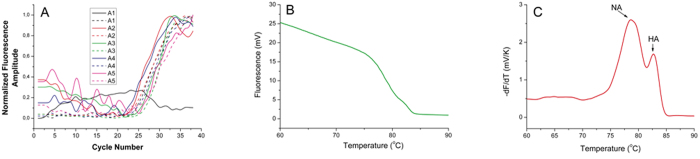
Amplification curves extracted for samples A1–A5 (referring to experiment codes in Table 2) at 68 °C (solid lines) and 76 °C (dashed lines). Sample A1 shows no amplification at 68 °C (black line) as expected (**A**), end point high resolution melting curve analysis of sample A2 after 40 cycles on the LightCycler (**B**–C)

This procedure was applied to a sample containing only a single amplicon (HA) with melting temperature of 76 °C (see Fig. 3A,B). We have then added to the sample a second amplicon (NA) with a melting temperature of 68 °C. A second peak corresponding to this amplicon can be observed in the respective MCA (Fig. 3C,D). Increasing the amount of NA added compared to HA lead to an even faster increase of the magnitude of peak at 68 °C (Fig. 3E,F). Extracting the fluorescence amplitude from the derived curves for each cycle at 68 °C and 76 °C respectively, results in two PCR amplification curves. Figure 4A shows the two amplification curves extracted for the experiments with a higher concentration of NA than HA (experiments A1 to A5 in Table 2).

The sample spiked with the cDNA of the HA gene yielded only a single amplification curve with melting temperature of 76 °C, which was expected. For all other experiments we were able to extract two amplification curves, one at a temperature of 68 °C and the second one at temperature of 76 °C. Threshold cycles (*C*_*T*_) were defined in the usual way. It was found that the difference in *C*_*T*_ (∆*C*_*T*_) of the two curves corresponded to the expected difference according to the ratio of concentrations of HA and NA. An overview of all conducted experiments is presented in Table 1. The results indicate that ∆*C*_*T*_ corresponds to the expected values. We can then conclude that the method can be used for multiplexed quantitative PCR. As an example, (see Table 1) sample A5 has the concentration of NA 19 times higher than the concentration of HA. With 100% PCR efficiency one would expect a ∆*C*_*T*_ of 4.25 (log_2_19). We have found the ∆*C*_*T*_value to be 4.0 ± 0.4 which is efficiency of 84%, reasonably close to the ideal value.

We have observed MCA peak broadening in our experiment due to applied conditions. It is necessary to run several replicates of the same experiment to suppress random errors and improve the result precision. The MCAs are recorded at a scanning rate of over 20 °C/s while the commercial systems for high resolution melting curve analysis (HRMCA)^17^ are usually operating with low temperature scan rates between 0.1 °C/s and 0.5 °C/s. Differentiation of amplicons with melting point differences *ΔT*_*m*_ as small as 1.2 °C have been demonstrated in HRMCA. However the method presented in this contribution requires greater *ΔT*_*m*_ due to peak broadening caused by the high heating rate.

We assume that a difference in amplicons melting temperatures’ of at least 5 °C would be sufficient to differentiate them from each other. This could be achieved by primer design since the melting temperature is a function of amplicon length and ratio of CG/AT content. Therefore an extension temperature of 60 °C and a denaturation temperature of 95 °C would allow for 6 different amplicons to be detected in the same droplet, assuming a *ΔT*_*m*_ of 5 °C.

Furthermore, the temperature measured by the sensor in HRMCA corresponds practically to the sample temperature due to the slow scanning rate. In our approach the sample temperature lags behind the temperature measured by the sensor underneath the sample^18^. This can be seen from the comparative end point HRMCA done with the LightCycler, Fig. 4B shows the fluorescence amplitude plotted as function of temperature with a temperature gradient of 0.1 °C/s. In Fig. 4C the first negative derivative of this curve with respect to temperature is shown, displaying two distinct peaks. Both melting temperatures found on the LightCycler are 8 °C higher than the temperatures we measured in our system. Nevertheless this *T*_*m*_ offset has no influence on quality of the results especially when this lag is known and the resulting temperature offset is calibrated.

Ideally the test should be performed with a clinical sample containing H7N9 virus starting with sample preparation followed with reverse transcription. VRC-based RT-PCR demonstrating almost all those steps was shown earlier, detecting both RNA from H5N1 avian influenza virus^19^ as well as from virus of severe acute respiratory syndrome (SARS)^20^. Processing clinical sample containing virus of avian influenza or SARS would also require laboratory classified as biosafety hazard level 3 which we do not have available. In previous work we developed the system based on spiking blood sample with RNA, here in this work we spiked PCR master mix with two types of cDNA.

## Conclusions

We have developed a method for quantitative, multi target PCR using only a single intercalating dye and thus only one fluorescence channel. Our approach allows researchers to perform affordable multiplex PCR using simple tools as well as reagents. Another advantage compared to conventional methods is small consumption of reagents due to small sample volume. Fluorescent amplitude sampling at rate above 100 samples per second is, however, required for constructing MCA during transition from annealing/extension to denaturation. This method might be particularly interesting for determination of serotype of virus such as dengue fever or an avian influenza. Other potential applications include measurement of viral loads in a sample, differentiation between H7N9 and H7N5, and quantitative detection of co-infections. We have experimentally verified the method using a homemade PCR system primarily for convenience as we have 100% control of the tool. In principle any commercial real-time PCR device would be capable of performing the same protocol as long as it has available fluorescent amplitude sampling rate of 1kHz or higher and can process samples with small volume of a 1 μL or below.

## Additional Information

**How to cite this article**: Ahberg, C. D. *et al.* Single Fluorescence Channel-based Multiplex Detection of Avian Influenza Virus by Quantitative PCR with Intercalating Dye. *Sci. Rep.* **5**, 11479; doi: 10.1038/srep11479 (2015).

## Figures and Tables

**Table 1:** Primers used in this study.

**Target gene**	**Primer name**	**Primer sequence (5’-3’)**	**Primer size (bp)**	**Amplicon size (bp)**
H7N9 HA	HA-Forward	TACAGGGAAGAGGCAATGCA	20	103
	HA-Reverse	AACATGATGCCCCGAAGCTA	20	
H7N9 NA	NA Forward	CCAGTATCGCGCCCTGATA	19	70
	NA Reverse	GCATTCCACCCTGCTGTTGT	20	

**Table 2:** Overview of conducted experiments, found threshold cycles (CT) for both melting temperatures as well as the difference between both threshold cycles.

**Experiment Code**	**Concentration of HA (ng/μL)**	**Concentration of NA (ng/μL)**	**CT at 68 °C (NA)**	**CT at 76 °C (HA)**	**Difference between CT at 68 °C and 76 °C**
A1	1.0*10^−07^	0	—	22.5	—
A2	5.0*10^−07^	5.0*10^−07^	22.8	22.4	0.4 ± 0.3
A3	3.2*10^−07^	6.4*10^−07^	25.8	24.7	1.0 ± 0.5
A4	9.0*10^−08^	9.1*10^−07^	23.7	20.9	2.8 ± 0.3
A5	5.0*10^−08^	9.5*10^−07^	25.7	29.5	4.0 ± 0.4
B1	0	1.0*10^−07^	27.5	—	—
B2	5.0*10^−07^	5.0*10^−07^	23.1	22.9	−0.1 ± 0.8
B3	6.4*10^−07^	3.2*10^−07^	23.4	24.6	−1.2 ± 0.1
B4	9.1*10^−07^	9.0*10^−08^	26.7	30.0	−3.3 ± 0.3
**B5**	9.5*10^−**07**^	5.0*10^−**08**^	**23.4**	**27.0**	**−3.5 **±** 0.1**
